# Tankyrases positively regulate influenza A virus replication via type I interferon response

**DOI:** 10.1128/jvi.01298-25

**Published:** 2025-10-02

**Authors:** Gayan Bamunuarachchi, Kishore Vaddadi, Yurong Liang, Zhengyu Zhu, Quanjin Dang, Sankha Hewawasam, Lin Liu

**Affiliations:** 1 Oklahoma Center for Respiratory and Infectious Diseases, Oklahoma State University 7618, Stillwater, Oklahoma, USA; 2 The Lundberg-Kienlen Lung Diseases and Infection Laboratory, Department of Physiological Sciences, Oklahoma State University 7618, Stillwater, Oklahoma, USA; Emory University School of Medicine, Atlanta, Georgia, USA

**Keywords:** influenza A virus, post-translational modifications, Poly (ADP-ribose) polymerases, TNKS1, TNKS2, type I interferon response

## Abstract

Influenza viruses are respiratory pathogens that cause both seasonal and unpredictable pandemic infections in humans. Currently approved anti-influenza drugs target the viral proteins. A recurrent mutation in the influenza genome leads to drug resistance and thus hinders the efficacy of anti-influenza drug treatments. Influenza A virus (IAV) depends on host factors to complete its virus life cycle. Thus, there are increasing interests in antiviral drugs that target host cellular proteins required for virus replication. Poly (ADP-ribose) polymerases (PARPs) are the host factors that modify protein functions by adding ADP-ribose to target proteins. Using the CRISPR activation system, we screened all 17 PARP members for its effects on IAV infection in lung epithelial A549 cells. Tankyrase 1 and 2 (TNKS 1/2 or PARP5A/5B) were found to be potent proviral factors. Knockout of TNKS1 or TNKS2 in HEK293T cells by CRISPR reduced viral mRNA and protein levels in cells and viral titers in culture media. Double knockout of TNKS1 and TNKS2 had a larger effect on IAV infection than single knockout of each isoform. The effect of TNKS double knockout on IAV replication was strain independent. Overexpression of TNKS1/2 in the double knockout cells restored the IAV replication to a level similar to the control cells. TNKS double knockout activated JNK/c-Jun signaling, enhanced Stat signaling, and increased type I interferon expression. Finally, *Tnks1* or *Tnks2* KO mice challenged with a sublethal IAV showed increased type I IFN response and reduced viral load in the lungs. The survival rate of *Tnks1* or *Tnks2* KO mice from a lethal IAV infection was significantly increased compared to wild-type mice. In conclusion, TNKS1 and TNKS2 regulate influenza virus infection via type I interferon response.

**IMPORTANCE:**

Poly (ADP-ribose) polymerases (PARPs) play a crucial role in DNA repair, cellular stress responses, epigenetics, gene transcription, and viral infections. However, the specific roles of PARPs in influenza A virus (IAV) infection remain unclear. In this study, we identified Tankyrase 1 and 2 (TNKS1/2 or PARP5a/5b) as the potent proviral factors. Knockout of TNKS1 or TNKS2 reduced viral replication *in vitro*, with the double knockout showing an even greater effect. TNKS double knockout also resulted in an increased type I interferon response to IAV infection. *In vivo*, Tnks1 or Tnks2 KO mice exhibited lower viral loads and higher survival rates following IAV challenge. Our findings highlight TNKS1/2 as important regulators of IAV infection and potential targets for antiviral therapies.

## INTRODUCTION

Influenza is a respiratory infectious disease transmitted through the respiratory route in humans. Mild influenza virus infection is confined to the upper respiratory tract. However, severe influenza virus infection can result in the spread of the virus into the lower respiratory tract and is often associated with secondary infection with bacteria, leading to pneumonia and acute respiratory failure. Global influenza virus infection is characterized by annual seasonal epidemics and unpredictable pandemics. The Centers for Disease Control and Prevention (CDC) estimated that influenza virus infection caused 9.31 million to 41 million illnesses, medical visits from 4.3 to 21 million, hospitalizations from 140,000 to 710,000, and deaths from 12,000 to 52,000 in the USA from 2010 to 2020 (1, 2). Age is a risk factor for influenza virus infection. Individuals who are very young (<1 year of age) and elderly (>65 years of age) are more susceptible to the virus. Other risk factors, such as chronic pulmonary and cardiac diseases, extreme obesity, and pregnancy, can also contribute to high mortality (3).

The influenza genome is segmented and comprises eight negative-sense single-stranded viral RNAs. This segmented genome encodes at least eleven viral proteins, including hemagglutinin (HA), neuraminidase (NA), matrix protein 1 (M1), matrix protein 2 (M2), nucleoprotein (NP), heterotrimeric RNA-dependent RNA polymerase (PA, PB1, PB2), nonstructural proteins 1 (NS1), nonstructural proteins 2 (NS2), and accessory protein PB1-F2 (4). NP, PA, PB1, and PB2 form a viral ribonucleoprotein (vRNP) complex that is associated with a viral RNA, which contains information needed for virus replication.

The primary site of influenza virus infection is the human respiratory tract, where HA proteins bind with sialic acid receptors on the epithelial cells to initiate the infection. Upon the binding, virions are internalized via endocytosis, followed by uncoating and releasing of vRNPs into the cytoplasm. vRNPs are then imported into the nucleus where the virus replication occurs. Newly synthesized viral mRNAs are exported into the cytoplasm and translated into viral proteins (5). Afterward, newly synthesized vRNPs and viral proteins are assembled into new virions and released from the cell membrane to complete the virus life cycle (4, 5).

Poly(ADP-ribose) polymerases (PARPs) are a specialized family of enzymes that transfer one or more ADP-ribose groups to target proteins and are evolutionarily conserved from bacteria to humans (6). The human genome encodes 17 PARPs, five being capable of catalyzing polyADP-ribosylation, 10 monoADP-ribosylation, and two catalytically inactive (6). All PARPs possess a catalytic domain known as the PARP domain. Some PARPs contain other structural domains, such as the ankyrin domain, a sterile alpha module (SAM) domain, and macrodomain, which are important for substrate binding and oligomerization.

ADP-ribosylation plays an important role in DNA repair, cellular stress responses, cell differentiation, energy metabolism, epigenetics, gene transcription, signal transduction pathways, and pathogen infections (6, 7). The PARP regulation of virus replication, such as coronavirus, herpes simplex virus 1 (HSV-1), Zika virus, Epstein-Barr virus, human cytomegalovirus (HCMV), and influenza virus, has been reported (8–13). PARPs can be either proviral or antiviral. PARP13 (ZAPL) is involved in ADP-ribosylation of influenza viral PA and PB1 proteins, which are further ubiquitinated and degraded by the ubiquitin-proteasome pathway (13). PARP1 is a proviral factor for influenza virus replication either by facilitating the efficient synthesis of viral NP or degrading host type I interferon receptor (14, 15).

Interferons (IFNs) serve as one of the first barriers the influenza virus must overcome to establish an infection. Type I IFNs such as IFNα and IFNβ play a major role in innate and adaptive immunity. Transcription activation of antiviral IFN-stimulated genes (ISG) by the type I IFN-Janus kinase (JAK)–signal transducer and activator of transcription (STAT) pathway is a key event in limiting virus replication (16). PARPs (PARP7, PARP10, PARP12L, and PARP14) have been identified as interferon-stimulated genes (ISGs) that are potent inhibitors of viral infections (9, 12, 17). For example, PARP7, PARP10, and PARP12L significantly inhibit the replication of Venezuelan equine encephalitis virus and other alphaviruses (17). Additionally, when activated by interferon signaling, PARP12 effectively prevents Zika virus replication (9). Moreover, PARP14 has been recognized as a crucial regulator of type I interferon production during coronavirus infections (12). Therefore, PARPs may exert their antiviral activity by acting as either ISG or type I IFN stimulators.

Tankyrase 1 (TNKS1) and Tankyrase 2 (TNKS2), also referred to as PARP5A and PARP5B, are highly conserved between humans and mice, sharing substantial sequence identity (>80%) and residing in syntenic chromosomal regions—hallmarks of true orthologs in the PARP gene family across mammalian species (7, 18–21). Both proteins contain three conserved domains: an ankyrin repeat domain that mediates protein–protein interactions, a sterile alpha motif (SAM) domain that may regulate oligomerization, and a carboxy-terminal PARP catalytic domain that is responsible for poly (ADP-ribosyl)ation activity (20). TNKS1 uniquely contains additional histidine–proline–serine (HPS)-rich domains (20). The ankyrin domain serves as a platform for numerous diverse protein binding partners, resulting in a wide range of biological activities. Both TNKS1/2 are present in the nucleus and cytoplasm and ubiquitously expressed across a variety of human tissues.

TNKS is involved in many biological processes, such as DNA repair, telomere length determination, cancer progression, lung fibrogenesis, and myelination (20). Several studies depict the role of TNKS in the replication and pathogenesis of viruses, such as human cytomegalovirus, Epstein-Barr virus, and herpes simplex virus (10, 11, 22). We have previously shown that miR-9-1 and miR-206 restrict influenza virus replication by targeting TNKS1 and TNKS2, respectively (23, 24). *Tnks*1 or *Tnks2* knockout (KO) mice do not show obvious phenotypes, and double KO of both isoforms is embryonically lethal, suggesting functional redundancy between the two isoforms (25, 26).

In this study, we performed CRISPR activation screening of human PARP family members for its effects on influenza A virus infection (IAV) and identified TNKS1 and TNKS2 as the potent proviral factors. Furthermore, KO of TNKS1 or TNKS2 in human cells attenuated IAV replication, and the double KO of both isoforms showed a larger effect than that of single KO. TNKS double KO showed an enhanced level of type I IFN responses in response to IAV infection. Finally, *Tnks*1 or *Tnks2* KO mice showed a significant increase in survival against a lethal IAV infection.

## MATERIALS AND METHODS

### Cell culture

Primary human bronchial tracheal epithelial cells (HBTEC) were purchased from Lifeline Cell Technology (Frederick, MD, USA). HBTEC cells were maintained in a T-75 flask with BronchiaLife Complete Medium (Lifeline Cell Technology, Cat # LL-0023) and used for the experiments until passage 8. Wild-type (WT), *TNKS1*, and *TNKS2* single KO and double KO (DKO) human embryonic kidney (HEK293T) cells, created using CRISPR technique, were kindly provided by Dr. Susan Smith (New York University School of Medicine) (27). These cells were cultured in DMEM media with 10% fetal bovine serum (FBS) and 1% penicillin and streptomycin (PS). Madin-Darby canine kidney epithelial (MDCK) cells were purchased from the American Type Culture Collection (ATCC, Manassas, VA, USA). The cells were maintained in DMEM media with 10% FBS and 1% PS in a 37°C cell incubator with a 5% CO_2_ supply, and fresh media was changed every other day.

### Viruses

Influenza virus H1N1 A/PuertoRico/8/34 (A/PR/8/34) strain was purchased from ATCC. Two strains of IAV H1N1 [A/Oklahoma/3052/2009 (pdm/OK/09) and H3N2 A/Oklahoma/309/2006 (A/OK/309/06)] were a gift from Dr. Gillian Air (University of Oklahoma Health Sciences Center). These viruses were propagated in the allantoic cavities of pathogen-free embryonated chicken eggs (10 days old) (Charles River Laboratories, MA, USA). Inoculated eggs were incubated at 35°C for 3 days. The allantoic fluid was harvested and centrifuged at 2,000 × *g* for 10 min. A hemagglutination assay was performed to estimate the level of virus production. Only the allantoic fluids that produced high titers were pooled and stored at −80°C.

### Influenza virus infection

WT and TNKS KO HEK293T cells (5 × 10^5^ cells/well) were seeded in collagen-coated 12-well plates and cultured for 24 h. Then, cells were infected with different IAV strains at an indicated multiplicity of infection (MOI) in a serum-free medium for 1 h. Subsequently, the inoculation media were replaced with fresh serum-free media supplemented with 0.3% bovine serum albumin (BSA) and 0.5 µg/mL L-(tosylamide-2-phenyl) ethyl chloromethyl ketone- (TPCK, Worthington Biochemical Corporation, Lakewood, NJ, USA)-treated trypsin (TPCK-trypsin). The cells were cultured for different times. Cells and media were used for the determination of viral mRNA and protein and virus titer, respectively.

### Real-time PCR

Total RNAs were isolated using 1 mL of TRI Reagent (Molecular Research Center, Cincinnati, OH, USA) according to the manufacturer’s protocol. One microgram (μg) of RNA was treated with RNase-Free DNase I (ThermoFisher Scientific, Waltham, MA, USA) for 30 min at 37°C in a thermocycler and reverse-transcribed into cDNA by using (M-MLV) reverse transcriptase (Thermo Fisher Scientific, Waltham, MA, USA), oligo (dT)18, deoxynucleoside triphosphate (dNTP), and random hexamer primers (Promega, Madison, WI, USA). Real-time PCR was performed in a 20 µL reaction containing diluted cDNA, gene-specific primers (Table S1), and SYBR Green PCR Master mMix (Eurogentec, Fremont, CA, USA) using ABI 7500 real‐time PCR System (Applied Biosystems, Foster City, CA, USA). β‐actin was used as the internal control to normalize the gene expression levels, and the expression level of a gene was calculated using the 2^−ΔCt^ method.

### CRISPR activation stable cell line selection

HEK293 cells were transduced with lentiviruses encoding dCas9-VP64 and MS2-P65-HSF1. After 48 h, the cells were subjected to selection with blasticidin (3 µg/mL) and hygromycin (60 µg/mL) for 7–10 days. Single colonies were then picked and expanded. Protein expression levels of dCas9-VP64 and MS2-P65-HSF1 in each colony were verified by western blotting using anti-Cas9 antibodies (1:1000, GTX-53807, GeneTex) and anti-NF-kB P65 antibodies (1:1000, Abcam, ab16502).

### Vector transfection and reporter assay

HEK293T cells were seeded in collagen-coated plates (6-, 12-, or 96-well) for 24 h and then transfected with TNKS1 and/or TNKS2 overexpression vectors (pLSJH-TNKS1 and pLSJH-TNKS2) (27) or reporter vectors for 24 h by using Lipofectamine 3000 (Invitrogen, Carlsbad, CA, USA).

#### Influenza virus reporter assay

An IAV inducible luciferase reporter vector (pHH21-NP-3′-UTR-Luc-NP-5′-UTR) was constructed previously (28), in which a firefly luciferase gene was inserted into RNA polymerase I expression vector pHH21, under the control of NP 5′ and 3′ UTRs of influenza A/WSN/33. WT and TNKS KO cells (4 × 10^4^ cells/well) were seeded into collagen-coated 96-well plates and co-transfected with pHH21-NP-3′-UTR-Luc-NP-5′-UTR (20 ng) and pRL-TK *Renilla* luciferase plasmid (5 ng). Twenty-four hours post-transfection, the cells were infected with A/PR/8/34, pdm/OK/09, and H3N2 A/OK/309/06 at an indicated MOI. Forty-eight hours post-infection (hpi), cells were lysed, and dual luciferase activities were measured by using the Dual-Luciferase Assay System kKt (Promega, Madison, WI, USA) and the FLUOstar OPTIMA microplate fluorometer (BMG LABTECH, Offenburg, Germany). Firefly luciferase activities were normalized to *Renilla* luciferase activities.

To determine the effect of PARPs (1-16) on IAV replication, HEK293-dCas9-VP64-MPH stable cells were transfected with a lenti-sgRNA-MS2-Zeo- PARPs (1-16) or non-template control sgRNA (NC) (100 ng), pHH21‐NP‐3′‐UTR‐LUC‐NP‐5′‐UTR (20 ng), and pRL‐TK *Renilla* plasmid (5 ng) using Lipofectamine 3000 (Invitrogen, USA). After 24 h, cells were then infected with pdm/OK/09 at an MOI of 0.05 for 48 h. The dual luciferase activities were measured as described above.

#### Pathway reporter assay

WT and TNKS KO cells (96-well plate, 4 × 10^4^ cells/well) were transfected with 60 ng interferon-stimulated response elements (ISRE) luciferase reporter plasmid (BPS bioscience, San Diego, CA, USA) or 50 ng of JNK luciferase reporter plasmid (QIAGEN, Germantown, MD, USA) for 24 h. Subsequently, cells were infected with A/PR/8/34 at an MOI of 0.01 for 24 h. Samples were collected, and dual luciferase activities were measured as mentioned above.

### Cell viability assay

Cell viability was assessed using the CellTiter-Blue Cell Viability Assay Kit (Promega, Madison, WI, USA) following the manufacturer’s protocol. Briefly, cells were seeded in 96-well plates and incubated with the CellTiter-Blue reagent at 37°C for 2 h. Fluorescence was then measured using a plate reader with excitation at 560 nm and emission at 590 nm.

### Western blotting

Western blot analysis was conducted as previously described (29). Proteins were separated on SDS-PAGE, transferred to nitrocellulose membrane, and incubated with the primary antibodies. The primary antibodies and dilutions are: rabbit anti-TNKS1 (1:1000, A302-399A, Bethyl Laboratories, TX, USA), rabbit anti-TNKS2 (1:1000, ab-155545, Abcam, Cambridge, UK), rabbit anti-Axin1 (1:1000, 2787S, Cell Signaling, Denver, MA, USA), mouse anti-NP (1:50, HB-65, ATCC), mouse anti-NS1 (1:1000, sc-130568, Santa Cruz, Dallas, TX, USA), rabbit anti-p-c-Jun (1:1000, CS-2361S, Cell Signaling), rabbit anti-c-Jun (1:1000, SC-1694, Santa Cruz), rabbit anti-Stat1 (1:1000, CS-14994S, Cell Signaling), rabbit anti-p-Stat1 (1:1000, CS-9167S, Cell Signaling), and mouse β-actin (1:1000, MA5-11869, Thermo Fisher Scientific). After incubating with primary antibodies, membranes were washed three times with Tris-buffered saline with 0.1% Tween-20 (TTBS), then incubated with horseradish peroxidase (HRP)-conjugated goat anti-rabbit or goat anti-mouse (1:1000) secondary antibodies for 1 h. Finally, membranes were washed (3× with TTBS), and protein bands were visualized using the SuperSignal West Pico PLUS chemiluminescent substrate (ThermoFisher Scientific). Intensities of the desired protein bands are densitometrically measured as previously described (30).

### Plaque assay

MDCK cells (1.5 × 10^5^/well) were seeded into 12-well plates and cultured for 24 h. The cells were washed with sterile PBS and inoculated with a series of tenfold dilutions of virus in a serum-free medium containing 1 µg/mL TPCK-trypsin for 1 h at 37°C. Simultaneously, 2× DMEM media containing 2 µg/mL TPCK-trypsin and 2% heated seaplaque agarose were acclimated in a 37°C water bath. After a 1-hour inoculation, virus-containing media was aspirated, and 2 mL of overlay media (1:1 ratio of 2× DMEM media containing 2 µg/mL TPCK-trypsin and 2% seaplaque agarose) were added to each well. Plates were kept at room temperature until the agarose solidified. Subsequently, plates were placed upside down at 37°C incubator with 5% CO_2_ for 4 days. The cells were fixed with 10% formaldehyde solution for 30 min. Overlay media were then removed, and the cells were stained with crystal violet solution for 2 min (Sigma, MO, USA). The excess staining solution was washed away under tap water. Finally, the plates were air-dried and used to determine the virus titers as previously described (31).

### Enzyme-linked immunosorbent assay (ELISA)

The secreted IFN-β in the culture media was measured by VeriKine Human IFN-β Sandwich ELISA Kit (PBL assay science, Piscataway, NJ). Briefly**,** 50 µL of IFN-β diluted standards or culture media were incubated with pre-coated microtiter wells for 1 h at room temperature. The samples were aspirated and washed with the washing buffer. Then, 100 µL of antibody solution was added and incubated for 1 h. Microtiter wells were washed and replaced with 100 µL of horseradish peroxidase-conjugated secondary antibody. After a 1-hour incubation at room temperature, wells were washed, and substrate solution (tetramethylbenzidine) was added. After a 15-minute incubation in the dark, the reaction was terminated by the addition of 100 µL of stop solution. Absorbance at 450 nm was read using a microplate reader (Bio-Rad, Hercules, CA, USA). IFN-β concentration was determined based on a standard curve.

### Animal studies

The breeding pairs of heterozygous *Tnks1*^+/-^ on C57BL/6NJ background and *Tnks2*^+/-^ on C57BL/6J background were obtained from Jackson Laboratory (Bar Harbor, ME, USA). Heterozygous mice were bred to generate homozygous *Tnks1*^-/-^ and *Tnks2*^-/-^ mice. These mice were maintained as homozygotes throughout this study. Wild-type (WT) mice that were matched with age, sex, and background to *Tnks1*^-/-^ and *Tnks2*^-/-^ mice were purchased from Jackson Laboratory.

#### Sublethal influenza virus infection

WT (C57BL/6NJ) and *Tnks1*^-/-^ or WT (C57BL/6J) and *Tnks2*^-/-^ mice were intranasally inoculated with 50 µL of H1N1 A/PR/8/34 (160 plaques forming unit [PFU] or 1× mouse lethal dose fifty [MLD_50_]). Mice were euthanized at 0, 3, or 7 days post-infection (dpi). The right lungs were lavaged to obtain bronchoalveolar lavage fluids, which were stored at −80°C for further use. The right lung tissues were snap-frozen in liquid nitrogen, powdered under liquid nitrogen by using mortar and pestle, aliquoted into three fractions, and stored at −80°C. Two fractions were used for mRNA and protein analyses, and the third fraction was dissolved in DMEM medium for virus titer determination. Left lungs were infused and fixed with 10% neutral buffered formalin (ThermoFisher Scientific) for tissue sectioning.

#### Lethal influenza infection

For survival studies, mice were intranasally infected with 2× MLD_50_ of H1N1 A/PR/8/34 (320 PFU in 50 µL). Mice were monitored for clinical signs and body weight loss daily for 14 days as previously described (24).

### Data analysis

A minimum of three independent experiments was used to analyze *in vitro* data. More than six animals per group were used for in *vivo* experiments. GraphPad Prism version 7.0 was used for statistical analysis. Student’s *t*-test and one-way or two-way ANOVA, followed by post hoc comparisons (Dunnett’s, Sidak’s, and Tukey’s pairwise comparisons), were used to compare two groups or multiple groups, respectively. A *P-*value of <0.05 was considered significant.

## RESULTS

### Screening of PARPs that regulate influenza virus infection

**Fig 1:**
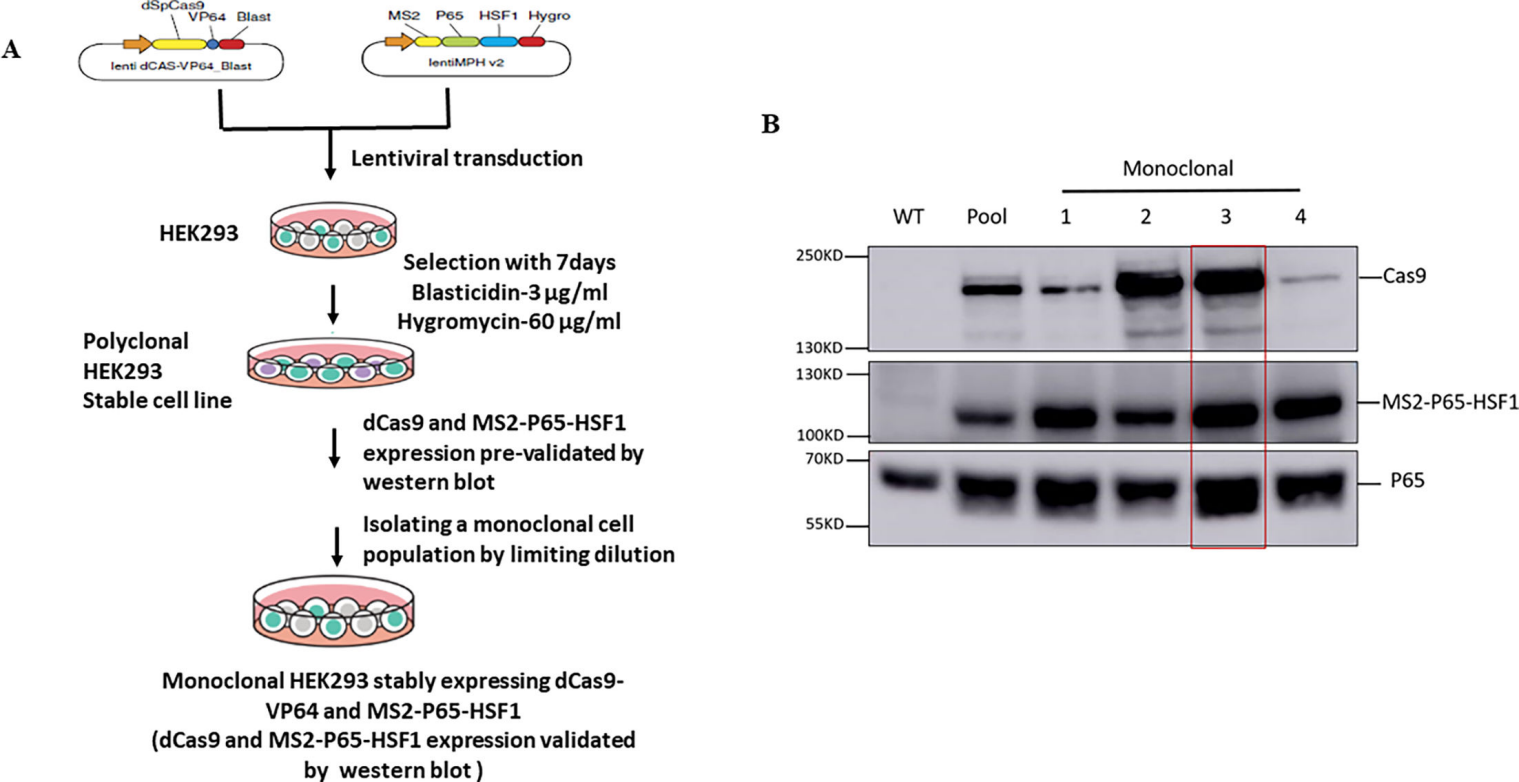
Establishment of CRISPR activation cell lines. (**A**) Schematic illustration of the generation of HEK293 cell lines stably expressing dCas9-VP64 and MS2-P65-HSF1. (**B**) Western blot analysis of whole-cell extracts from the initial polyclonal stable cells (pool) and individual cell clones (monoclonal, 1–4) using anti-Cas9 and anti-P65 antibodies. Wild-type HEK cells (WT) were used as a control.

To identify which PARPs regulate IAV infection, we employed a CRISPR activation (CRISPRa) screen (32). CRISPRa is a modification of the CRISPR/Cas9 system that enhances gene expression by directing specific transcriptional activators to target promoters. This approach has a key advantage over traditional cDNA overexpression systems, i.e., its ability to activate large transcripts and to regulate all the transcripts produced from a single gene promoter, enabling more comprehensive gene activation studies (33). To perform CRISPR activation of PARP screening, we first generated a polyclonal CRISPRa stable cell line and isolated monoclonal lines by limited dilution (Fig. 1A). We selected monoclonal cell line 3 with the highest transgene expression for further experiments (Fig. 1B).

To activate the expression of each PARP member, we selected three independent sgRNAs per gene, targeting sequences located within 200 bp upstream of the transcription start site (TSS) of the canonical RefSeq transcript, following the optimized approach described by Konermann et al. (33). These sgRNAs do not target internal exons or alternative promoter regions, which reduces the likelihood of unintended isoform activation. None of these sgRNAs overlap with known alternative transcript regions based on current genomic annotations. sgRNA chromosomal coordinates and target sequences are listed in Table S3.

To determine the mRNA levels of each PARP, we designed real-time primers that span exons uniquely present in the full-length transcripts of PARPs. These primer sets were carefully designed to avoid regions shared with known splice isoforms or related PARP family members. Each primer set was further validated by NCBI BLASTN to ensure specificity to the intended target and absence of cross-reactivity with other PARP transcripts. The complete list of primers used for all 16 PARPs is provided in Table S1.

**Fig 2:**
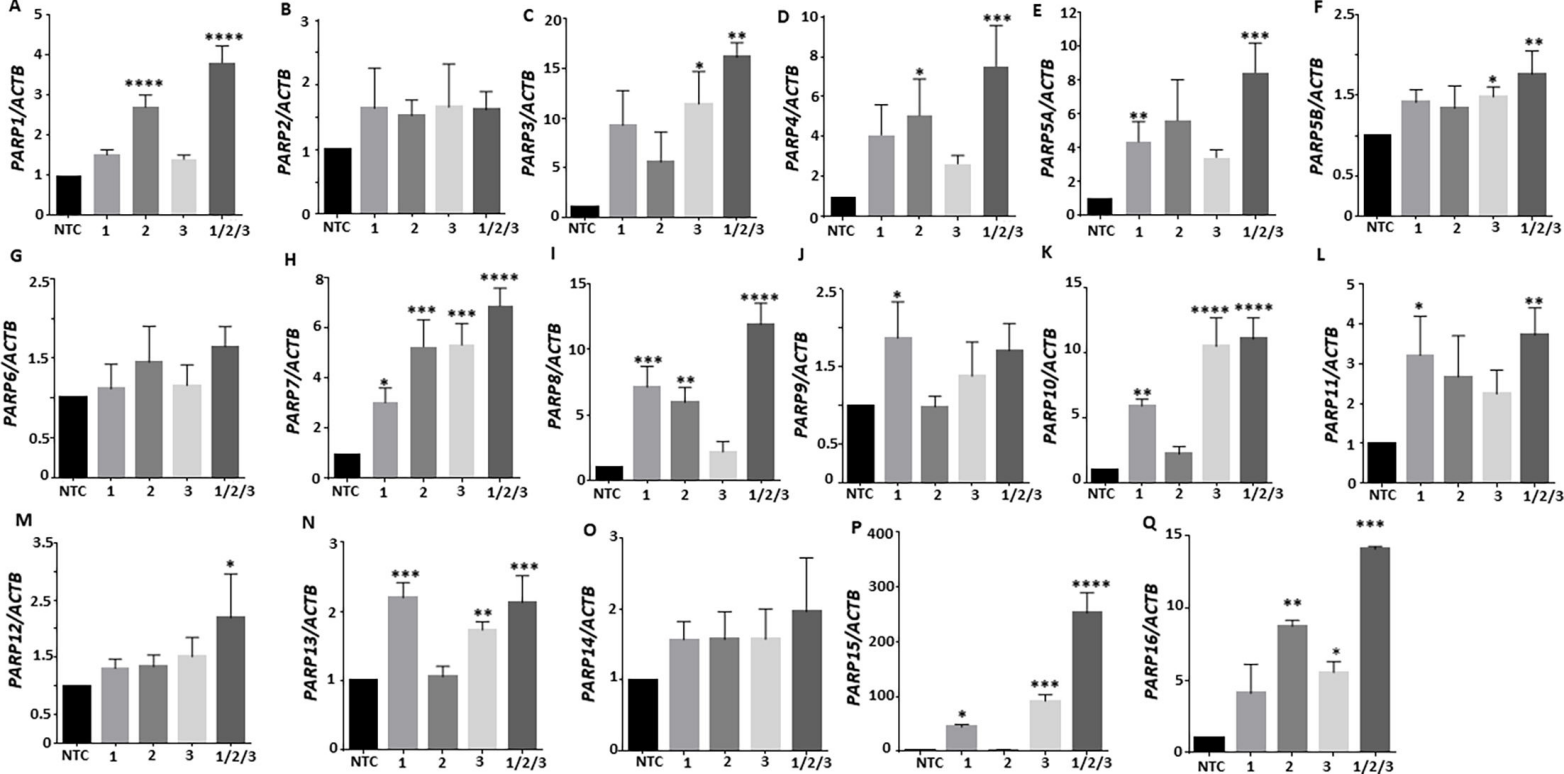
CRISPR activation of PARPs. HEK293-dCas9-VP64-MPH stable cells (clone 3) were transfected with a lenti-sgRNA-MS2-Zeo- PARPs (1-16) sgRNAs 1, 2, 3, and 1/2/3 pooled or non-template control sgRNA (NTC) for 24 h. (**A–Q**) The relative mRNA expression level of PARPs (1-16) was determined by real-time PCR. Data are expressed as the mean ± SE of three independent experiments. *P < 0.05, **P < 0.01, ***P < 0.001, ****P < 0.0001 versus NTC-sgRNA (one‐way ANOVA, followed by Dunnett’s multiple comparisons test).

HEK293-dCas9-VP64-MPH stable cells (clone #3) were transfected with each individual sgRNA or the combination of three sgRNAs, and real-time PCR was used to detect the mRNA expression of PARPs. In general, the combination of three sgRNAs was more effective in increasing gene expression compared to individual sgRNAs (Fig. 2). All the *PARP* mRNA expression levels were increased by the three sgRNAs except PARP2, PARP6, PARP9, and PARP14, which showed an increasing trend but did not reach a significant level. *PARP15* mRNA had the highest fold change of >200, caused by three sgRNAs, while PARP3, PARP8, PARP10, and PARP16 mRNA had over a tenfold change.

**Fig 3:**
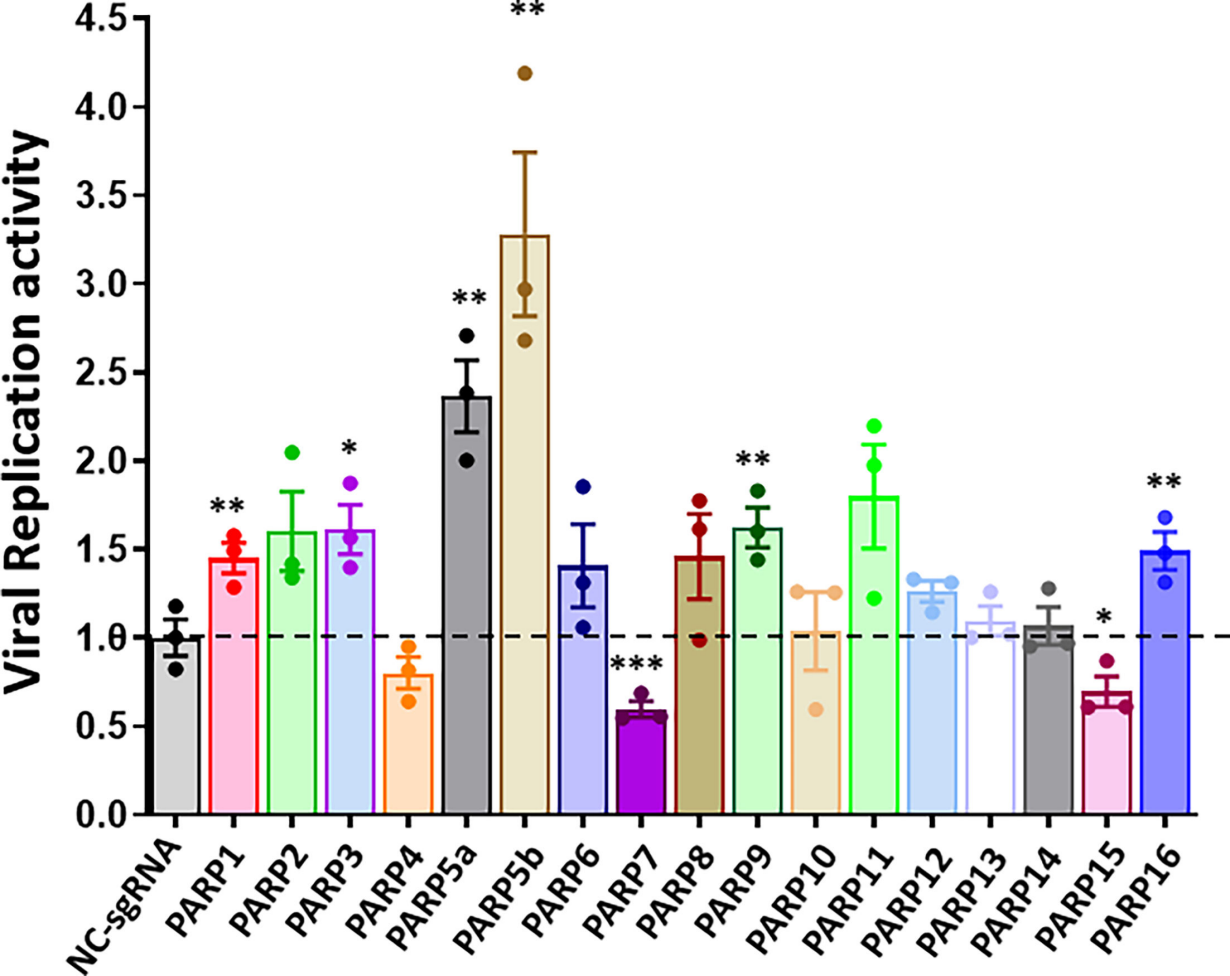
Effects of CRISPR activation of PARPs on IAV replication. HEK293-dCas9-VP64-MPH stable cells were transfected with a combination of three lenti-sgRNA-MS2 targeting each of PARPs or non-template control sgRNA (NTC) (100 ng), the IAV firefly luciferase reporter plasmid vNP‐luc/pHH21 (20 ng), and pRL‐TK *Renilla* plasmid (5 ng) for 24 h The cells were then infected with influenza 2009 pandemic clinic isolate A/Oklahoma/3052/09 (pdm/OK/09) H1N1 at an MOI of 0.05 for 48 h. Firefly luciferase activity was normalized to pRL‐TK *Renilla* luciferase activity. The results are expressed as a ratio to NTC‐transfected cells. Data are the mean ± *SE* of three independent experiments. *P < 0.05, **P < 0.01, and ***P < 0.001 vs. NTC (Student’s *t*‐test).

We next determined the effects of increased PARP levels by CRISPRa on IAV infection using an IAV luciferase reporter assay (24). The IAV luciferase reporter vector contains a firefly luciferase gene flanked with the 5′- and 3′-untranslated regions of WSN NP, through which the level of virus replication in the cells can be measured. The reporter vector was transfected into HEK293 cells stably expressing dCas9-VP64 and MS2-P65-HSF1, along with the lenti-sgRNA-MS2-Zeo-PARPs (1-16) sgRNA 1, 2, 3, or non-template control sgRNA (NTC). The cells were then infected with pdm/OK/09 at an MOI of 0.05 for 48 h, followed by a dual luciferase assay. The induced expression of PARP1, PARP3, PARP5a, PARP5b, PARP9, and PARP16 by CRISPRa increased the reporter activity (Fig. 3), suggesting their pro-viral activity. In contrast, enhanced expression of PARP7 and PARP15 decreased the reporter activity, indicating their antiviral activity. Given that TNKS1 and TNKS2 (PARP5a and PARP5b) had the most significant effects in IAV infection, we selected these two genes for further studies.

### Influenza virus infection induces TNKS1 and TNKS2 expression

**Fig 4:**
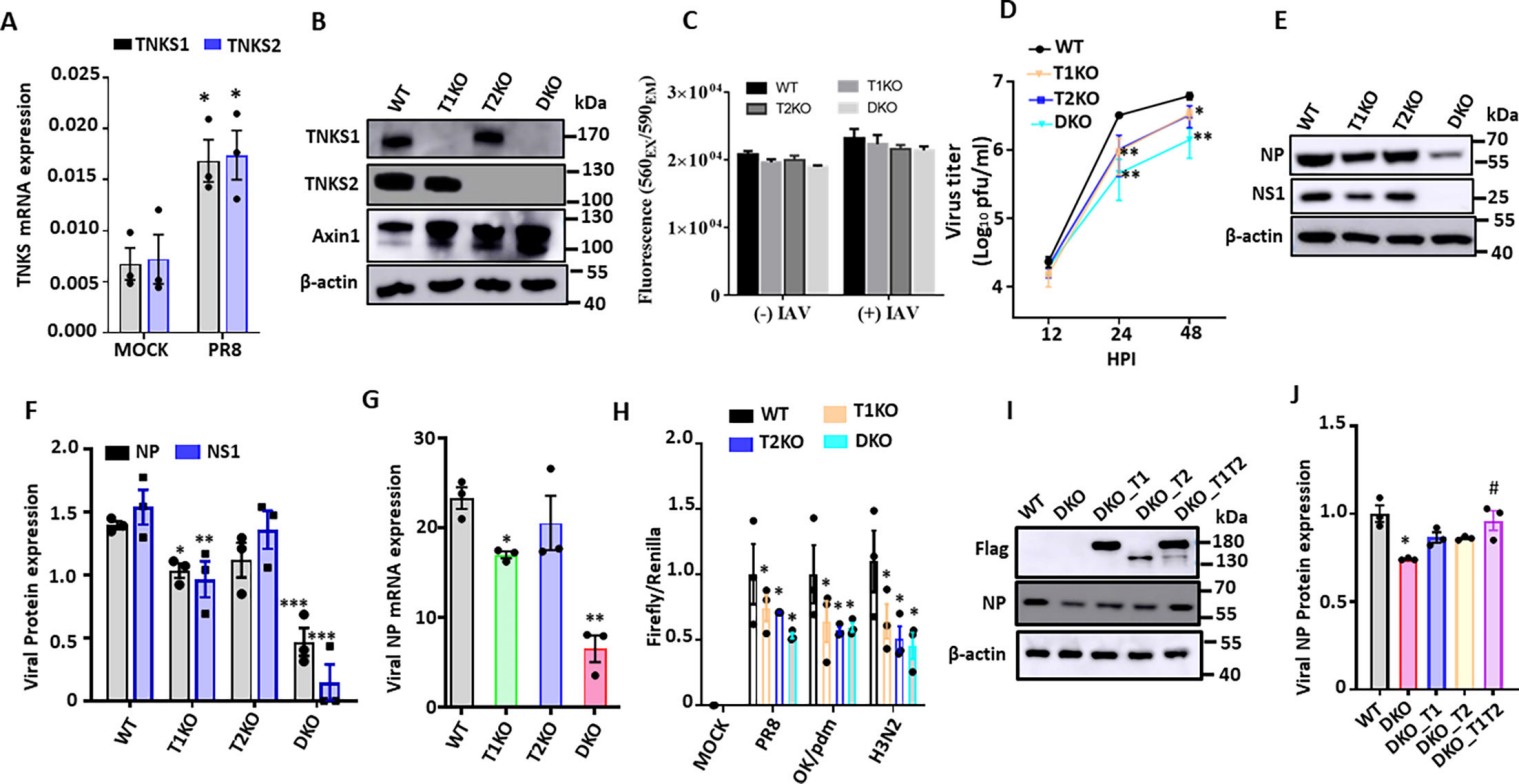
TNKS knockout represses IAV replication. (**A**) HBTECs were infected with H1N1 A/PR/8/34 at an MOI of 0.1 and samples were collected at 24 hpi. The mRNA expression levels of TNKS1 and TNKS2 were measured by real-time PCR and normalized to β-actin. (**B**) TNKS1, TNKS2, and Axin1 protein levels of HEK293T cells with wild type (WT), TNKS1 KO (T1KO), TNKS2 KO (T2KO), and TNKS1 and TNKS2 double KO (DKO) as determined by western blotting. (**C**) Cell viability was measured in uninfected cells (-IAV) and PR8 (MOI, 0.01; 48 h)-infected cells (+IAV) by CellTiter Blue assay. (**D–G**) WT and TNKS KO cells were infected with A/PR/8/34 at an MOI of 0.01, and samples were collected at 0, 24, and 48 hpi. Virus production in the culture media at 0, 24, and 48 hpi was titrated by plaque assay (**D**). Viral NP and NS1 protein levels at 48 hpi were measured by western blotting and normalized to β-actin (**E, F**). Viral NP mRNA expression at 48 hpi was measured by real-time PCR and normalized to β-actin (**G**). (**H**) WT and TNKS KO cells were transfected with the IAV firefly luciferase reporter plasmid vNP-luc/pHH21 (20 ng) and pRL-TK *Renilla* plasmid (5 ng) for 24 h. The cells were then infected with A/PR/8/34, pdm/Ok/09, or H3N2 A/OK/309/06 at an MOI of 0.01 for 48 h. Firefly luciferase activities were normalized to *Renilla* luciferase activity and further normalized to WT. (**I, J**) WT and DKO were transfected with control plasmid (1,000 ng pLSJH-Con) and TNKS1 expression plasmid (500 ng pLSJH-TNKS1 and 500 ng pLSJH-Con) (DKO-T1), TNKS2 expression plasmid (500 ng pLSJH-TNKS2 and 500 ng pLSJH-Con) (DKO-T2), or TNKS1 and TNKS2 expression plasmids (500 ng pLSJH-TNKS1 and 500 ng pLSJH-TNKS2) for 24 h. The cells were then infected with A/PR/8/34 at an MOI of 0.01 for 48 h. TNKS1 and TNKS2 expression and viral NP expression were determined by western blotting using anti-flag and anti-NP antibodies, respectively. NP protein expression was densitometrically analyzed and normalized to β-actin. The results of three independent experiments are displayed as the mean ± SE. (**A**) *P < 0.05 vs. Mock, Student’s *t-test*. (**D, F, H**) *P < 0.05, **P < 0.01, ***P < 0.001 vs. WT, ^#^*P* < 0.05 vs. DKO, two-way ANOVA, followed by Sidak’s pairwise comparison. (**G, J**) *P < 0.05, ^#^*P* < 0.05 vs. DKO vs. one-way ANOVA followed by Dunnett’s pairwise comparison*.*

We have previously shown that influenza virus infection increased the expression of TNKS1 and TNKS2 in lung epithelial A549 cancer cell line and HEK293 cells (23, 24). We further evaluated the TNKS expression in primary lung epithelial cells (HBTECs) infected with A/PR/8/34 at an MOI of 0.1 for 24 h. *TNKS1* and *TNKS2* mRNAs were also upregulated in human primary cells by IAV infection (Fig. 4A).

### TNKS knockout attenuates influenza virus replication

To confirm the effects of TNKS1 and TNKS2 on influenza virus infection, we used TNKS single and double isoform KO in HEK293 cells (TNKS1 KO, TNKS2 KO, and DKO) that were established using the CRISPR technique by Bhardwaj et al. (27). We confirmed the deletions of TNKS1 and TNKS2 proteins in these cells (Fig. 4B). TNKS is a well-known negative regulator of Axin1. Axin1 protein expression was increased in single and double KO cells with the highest induction in double KO cells (Fig. 4B). The cell viability of KO cells was measured before and after A/PR/8/34 infection (MOI, 0.01, 48 h) by the CellTiter-Blue assay. Compared to WT, TNKS1 KO, TNKS2 KO, and DKO did not show any significant differences in cell viability under both conditions (Fig. 4C), suggesting that TNKS single and double isoform deletions do not affect the cell viability.

Next, we determined virus growth kinetics in the KO cells infected with an MOI of 0.01 of A/PR/8/34 by measuring virus production in culture media using a plaque assay. Starting from 24 hpi, all three TNKS KO cells showed a significant reduction in virus titer compared to WT cells, with a bigger decrease in DKO cells (Fig. 4D). Viral NP and NS1 proteins were reduced 26 ± 4% and 38 ± 8% in TNKS1 KO cells at 48 hpi, respectively (Fig. 4E and F). A trend of reduction in NP and NS1 protein levels was observed in TNKS2 KO cells, but the effect was not significant. Similar to virus titer, DKO cells showed the highest decreases in NP (67 ± 7%) and NS1 (90 ± 19%) protein levels (Fig. 4E and F). These effects were also observed for viral NP mRNA expression (Fig. 4G).

Using an IAV inducible reporter assay (24), we determined the effects of TNKS KO on the infection of different strains of IAV. TNKS KOs exhibited reduced replication of several IAV strains, including H1N1 A/PR/8/34, A/Oklahoma/3052/09 (2009 pandemic strain) and H3N2 A/OK/309/06 (Fig. 4H). In contrast to other assays, we did not observe significant differences between single KOs and double KOs.

Finally, we examined whether ectopic expression of TNKS1 and/or TNKS2 in DKO cells using Flag-tagged TNKS1 and/or TNKS2 expression vectors could reverse the effects of TNKS1 and TNKS2 KO on influenza virus infection. Ectopic expression levels of TNKS1 and TNKS2 were confirmed by western blot analysis using anti-flag antibodies (Fig. 4I). The viral NP protein level decreased in DKO cells compared to WT cells. Ectopic expression of both TNKS1 and TNKS2 in DKO cells restored the viral NP protein level to that in WT (Fig. 4I and J). Although the ectopic expression of TNKS1 and/or TNKS2 in DKO cells partially restored viral NP protein levels, the rescue did not reach wild-type levels. This incomplete restoration may be due to interference by the N-terminal FLAG tag with proper protein function or localization. Furthermore, ectopic expression from a strong promoter may not replicate the endogenous timing, regulation, or expression level of TNKS1/2, which could be necessary for full functional activity during viral infection. Collectively, these findings suggest that both TNKS1 and TNKS2 are pro-viral factors against IAV. Moreover, double KO of TNKS1 and TNKS2 showed a bigger effect on IAV infection than that of single KO.

### TNKS DKO increases type I IFN response

**Fig 5:**
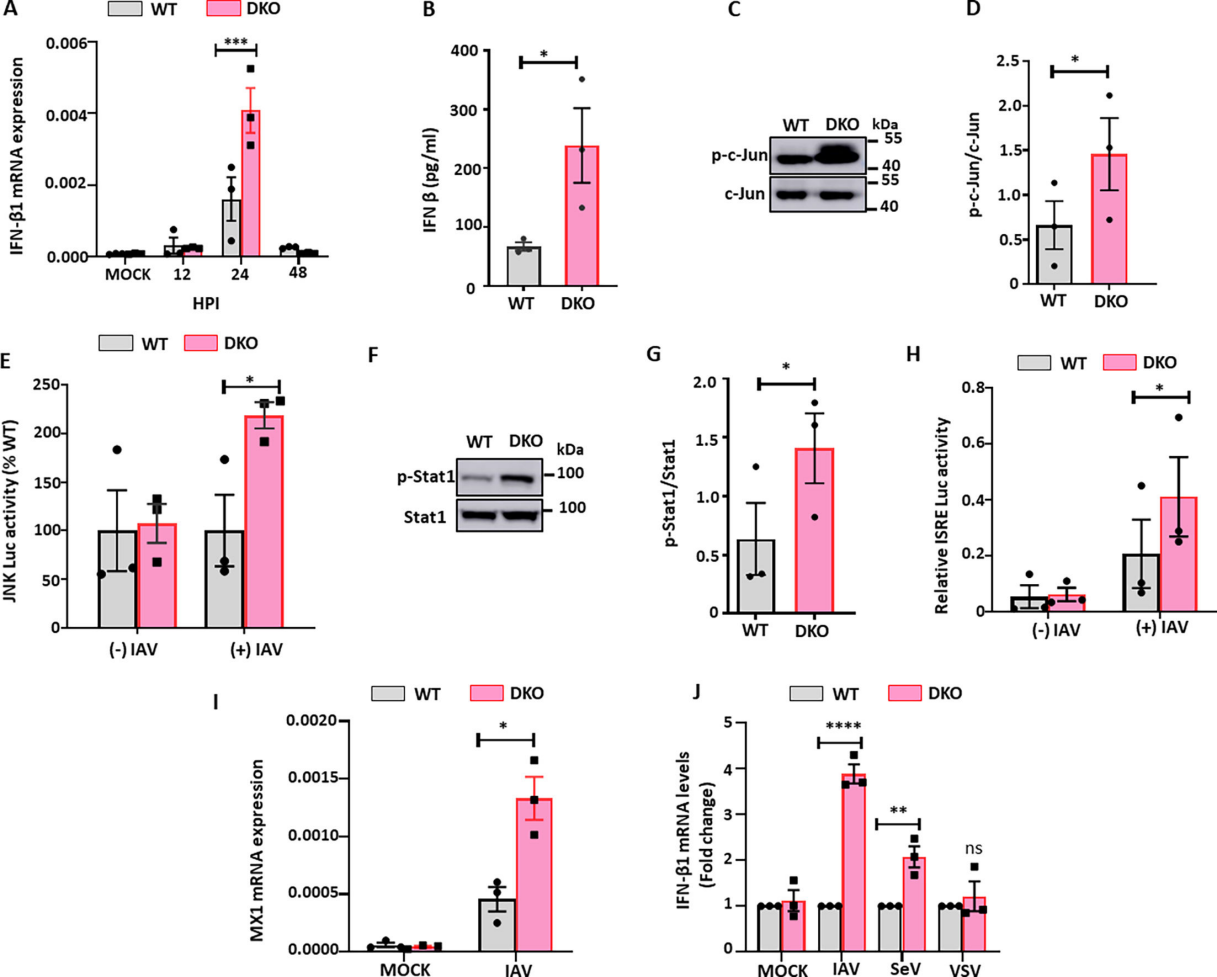
DKO enhances type I IFN response *in vitro*. (**A–B**) WT and TNKS DKO cells were uninfected (MOCK) or infected with A/PR/8/34 (MOI = 0.01) for 12, 24, and 48 h. (**A**) The mRNA expression levels of IFN-β1 were determined by real-time PCR and normalized to β-actin. (**B**) IFN-β secretion in the culture media at 24 hpi was measured by ELISA. (**C, D**) WT and TNKS DKO cells were infected with A/PR/8/34 (MOI = 0.01) for 24 h. The protein expression levels of p-c-Jun were determined by western blotting and expressed as a ratio to c-Jun. (**E**) WT and DKO cells were transfected with JNK Luc vector for 24 h and then uninfected (-IAV) or infected (+IAV) with A/PR/8/34 (MOI = 0.01) for 24 h. Firefly luciferase activities were normalized to *Renilla* luciferase activities. The results were expressed as a percentage of WT. (**F, G**) The protein expression levels of p-Stat1 and Stat 1 at 24 hpi were determined by western blotting, and the results were expressed as a ratio of p-Stat1 to Stat1. (**H**) WT and DKO cells were transfected with the ISRE-Luc reporter vector for 24 h and then uninfected (-IAV) or infected (+IAV) with A/PR/8/34 (MOI = 0.01) for 24 h. *Firefly* and *Renilla* luciferase activities were measured and normalized to *Renilla* luciferase activities. (**I**) MX1 mRNA levels were determined in WT and TNKS DKO cells uninfected (MOCK) or with A/PR/8/34 (MOI = 0.01) for 24 h by real-time PCR and normalized to β-actin. (**J**) WT and TNKS DKO cells were infected with A/PR/8/34 (MOI = 0.1), vesicular stomatitis virus (MOI = 0.1), and Sendai virus (MOI = 0.1) for 24 h. The mRNA expression levels of IFN-β1 were determined by real-time PCR and normalized to β-actin. The results of three independent experiments are displayed as the mean ± SE. *P < 0.05, **P < 0.01, ***P < 0.01, and ****P < 0.0001 vs. WT. (**A, J**) Two-way ANOVA followed by Sidak’s pairwise comparison. (**B, D, E, G, H, I**) Student’s *t-test*.

Because TNKS DKO cells showed a significant reduction in viral replication (Fig. 4), we selected DKO cells to further investigate antiviral mechanisms. Host cells mount early and robust responses that prime cells for an antiviral state via the type I IFN pathway. To determine whether TNKS DKO cells enhance this response, we assessed the expression of type I IFN pathway genes. WT and TNKS DKO cells were infected with A/PR/8/34 at an MOI of 0.01, and the mRNA expression of IFN-β1 was measured by real-time PCR. At 24 hpi, DKO cells exhibited higher IFN-β1 mRNA expression compared to WT cells (Fig. 5A). ELISA analysis revealed that the IFN-β production in the culture media of DKO cells increased 3.5-fold at 24 hpi (Fig. 5B).

During viral infections, Jun kinase (JNK)-dependent activation of IRF3 and/or c-Jun is one of the pathways that induces type I IFN expression (34, 35). The phosphorylation of c-Jun results in its nuclear translocation and the activation of activator protein 1 (AP-1), a transcription factor important in IFN-β gene expression (36). Therefore, we examined the effects of TNKS DKO on JNK/c-Jun signaling. Compared to WT cells, TNKS DKO cells showed higher levels of c-Jun phosphorylation (Fig. 5C and D) and increased JNK/AP-1 luciferase reporter activity by 218 ± 12% (Fig. 5E).

Type I IFNs induce IFN-stimulated genes (ISGs) expression via Stat1 signaling, and the phosphorylation of Stat1 is key to activate this signaling pathway. Therefore, we examined the effects of TNKS DKO on the phosphorylation of Stat1 during IAV infection. DKO enhanced the phosphorylation of Stat1 (Fig. 5F and G). The phosphorylation of Stat1 leads to the dimerization of Stat1 and Stat2. The complex then translocates into the nucleus and binds the interferon-stimulated response elements (ISRE) to induce the expression of ISGs. The transcriptional activity of ISRE, as measured by ISRE luciferase reporter assay, was increased twofold in IAV-infected DKO cells compared to WT (Fig. 5H). Furthermore, DKO cells exhibited higher mRNA expression of the interferon-stimulated gene MX1 compared to WT cells (Fig. 5I).

To assess whether the enhanced interferon signaling observed in TNKS-deficient cells is virus-specific, we measured IFN-β1 expression following infection with different RNA viruses: influenza A virus (IAV)**,** Sendai virus (SeV), and vesicular stomatitis virus (VSV). Compared to WT cells, DKO cells exhibited significantly increased IFN-β1 expression upon IAV and SeV infection**,** but not in response to VSV (Fig. 5J). These findings suggest that TNKS-mediated suppression of type I interferon responses is virus-specific and may depend on the particular innate immune pathways activated by each pathogen.

### TNKS regulates influenza virus infection via Axin1

**Fig 6:**
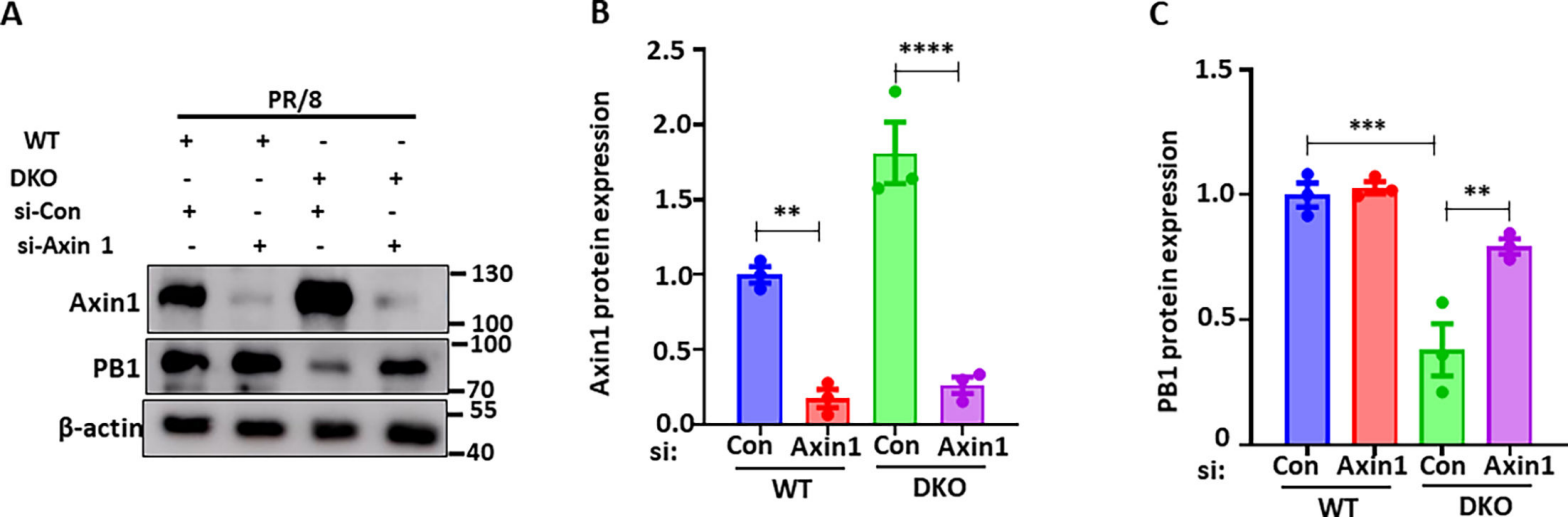
TNKS KO reduces IAV infection through Axin1. WT and TNKS DKO cells were transfected with control (siCon) and Axin1 siRNA (siAxin1) and infected with A/PR/8/34 (MOI = 0.01) for 48 h. Western blotting was performed to determine Axin1 and PB1 protein levels (**A**). The relative amounts of Axin1 (**B**) and viral protein PB1 (**C**) levels were normalized to β-actin. Data were presented as the mean ± SE of three independent experiments. **P < 0.01, ***P < 0.001, and ****P < 0.0001 (one‐way ANOVA, followed by Tukey’s multiple comparisons test).

TNKS has been reported to negatively regulate Axin1 (37). We have previously shown that Axin1 is downregulated by influenza virus infection, and overexpression of Axin1 inhibits influenza virus infection (36). As shown in Fig. 4B, TNKS single and double KO cells showed increased Axin1 protein expression, with DKO being the highest. To determine whether the effects of TNKS on IAV infection are via Axin1, we knocked down Axin1 expression in WT and DKO cells by using short interfering RNA (siRNA). siAxin1 transfection significantly reduced Axin1 protein expression in WT and DKO cells (Fig. 6A and B). In siRNA control cells, TNKS DKO cells had reduced PB1 expression compared to WT. However, this effect was no longer observed in Axin1 knockdown cells (Fig. 6A and C), suggesting that TNKS regulates IAV infection via Axin1.

### The deletion of TNKS1 or TNKS2 reduces IAV replication in mice

**Fig 7:**
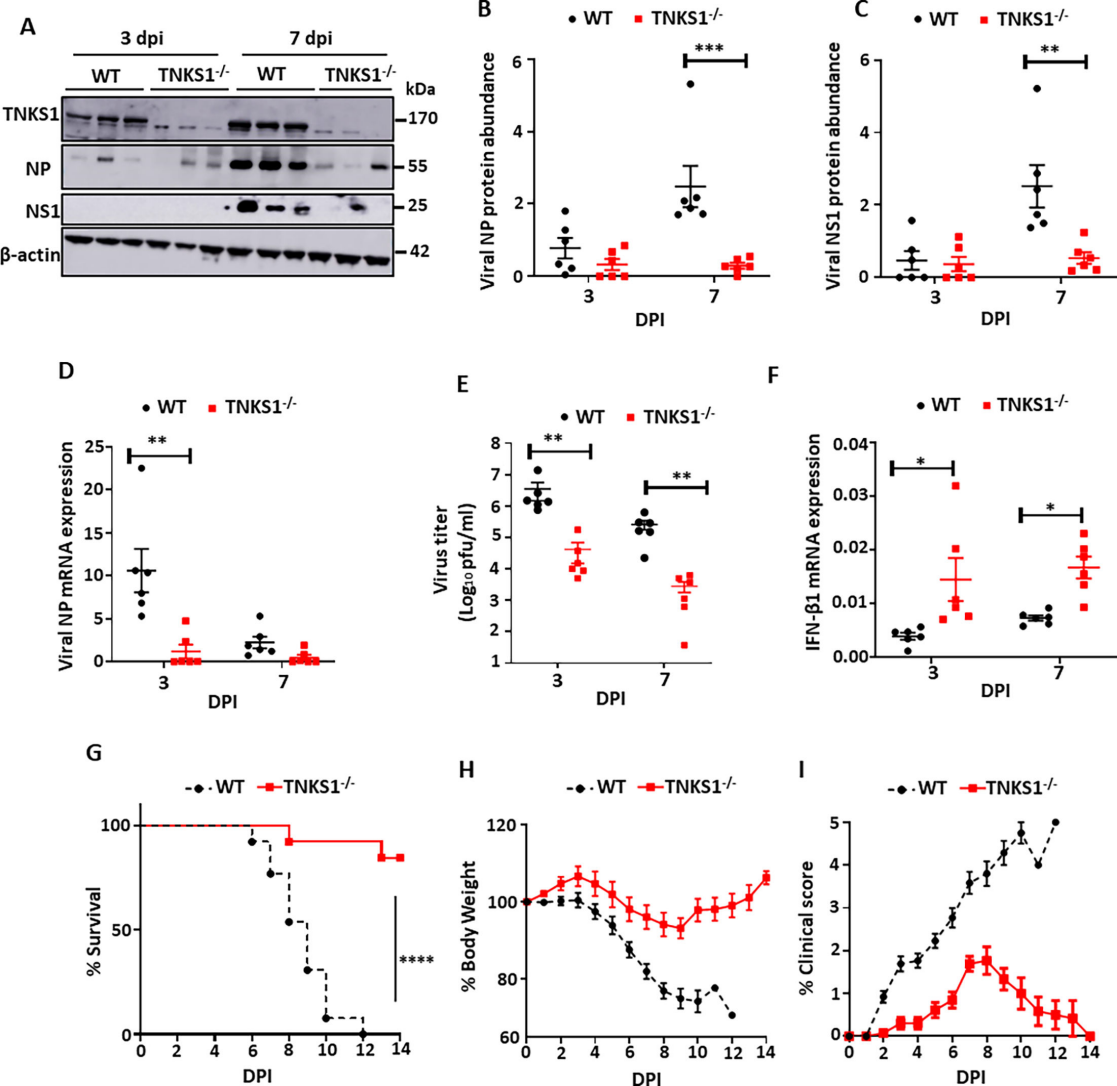
TNKS1 KO in mice attenuates IAV replication and increases survival. (**A–F**) WT and *Tnks1*^-/-^ mice (*N* = 6, 3 males and 3 females per group) were infected intranasally with a sublethal dose of A/PR/8/34 (160 PFU/mouse). Lung tissue samples were collected at 3 and 7 dpi. TNKS1, NP, and NS1 protein levels were measured by western blotting and normalized to β-actin (**A, B, C**). NP and IFN-β1 mRNA expression was measured by real-time PCR and normalized to β-actin (**D, F**). Virus production in the lungs was measured by plaque assay (**E**). (**G–H**) WT and *Tnks1*^-/-^ mice (*N* = 13, 8 males and 5 females per group) were infected intranasally with a lethal dose of A/PR/8/34 (320 PFU/mouse, 2 × MLD_50_). Kaplan-Meier survival analysis of mice (**G**). Body weight loss and clinical signs were measured daily (**H, I**). The results were displayed as the mean ± SE. **P < 0.01, ***P < 0.001, ****P < 0.0001 vs. WT. (**B–F**) Two-way ANOVA followed by Sidak’s pairwise comparison. (**G**) Mantel-Cox test with Bonferroni-corrected threshold.

TNKS DKO mice are embryonically lethal (26). We utilized TNKS1 or TNKS2 KO (*Tnks1*^-/-^ and *Tnks2*^-/-^) mice to evaluate the effects of TNKS1 or TNKS2 on IAV infection *in vivo*. We first studied *Tnks1*^-/-^ mice. WT and *Tnks1*^-/-^ mice were infected with a sublethal dose of A/PR/8/34 (160 pfu) at day 0. Lung tissues were collected at 3 and 7 dpi. TNKS1 and viral protein expression levels were determined by western blot. The deletion of TNKS1 was confirmed in the lungs of *Tnks1*^-/-^ mice (Fig. 7A). Viral NP and NS1 protein levels in *Tnks1*^-/-^ mice were decreased by 89 ± 8% and 79 ± 18%, respectively, at 7 dpi (Fig. 7A through C). Furthermore, the mRNA expression of viral NP in the lungs of *Tnks1*^-/-^ mice was also reduced at 3 dpi (Fig. 7D). *Tnks1*^-/-^ mice showed 2- and 1.5-fold log reduction in viral titers in the lungs at 3 and 7 dpi, respectively (Fig. 7E). IFN-β1 mRNA expression was increased at both 3 and 7 dpi in the lungs of *Tnks1*^-/-^ mice compared to these in WT mice (Fig. 7F).

Next, we performed a survival study to evaluate whether the deletion of TNKS1 protects mice from a lethal influenza virus infection. WT and *Tnks1*^-/-^ mice were infected with 2 × MLD_50_ of mouse-adapted H1N1 A/PR/8/34 and monitored for body weight loss, clinical signs, and survival. *Tnks1*^-/-^ mice significantly improved mouse survival against a lethal IAV infection (Fig. 7G). *Tnks1*^-/-^ mice lost less body weight and had lower clinical scores compared to WT mice (Fig. 7H and I).

**Fig 8:**
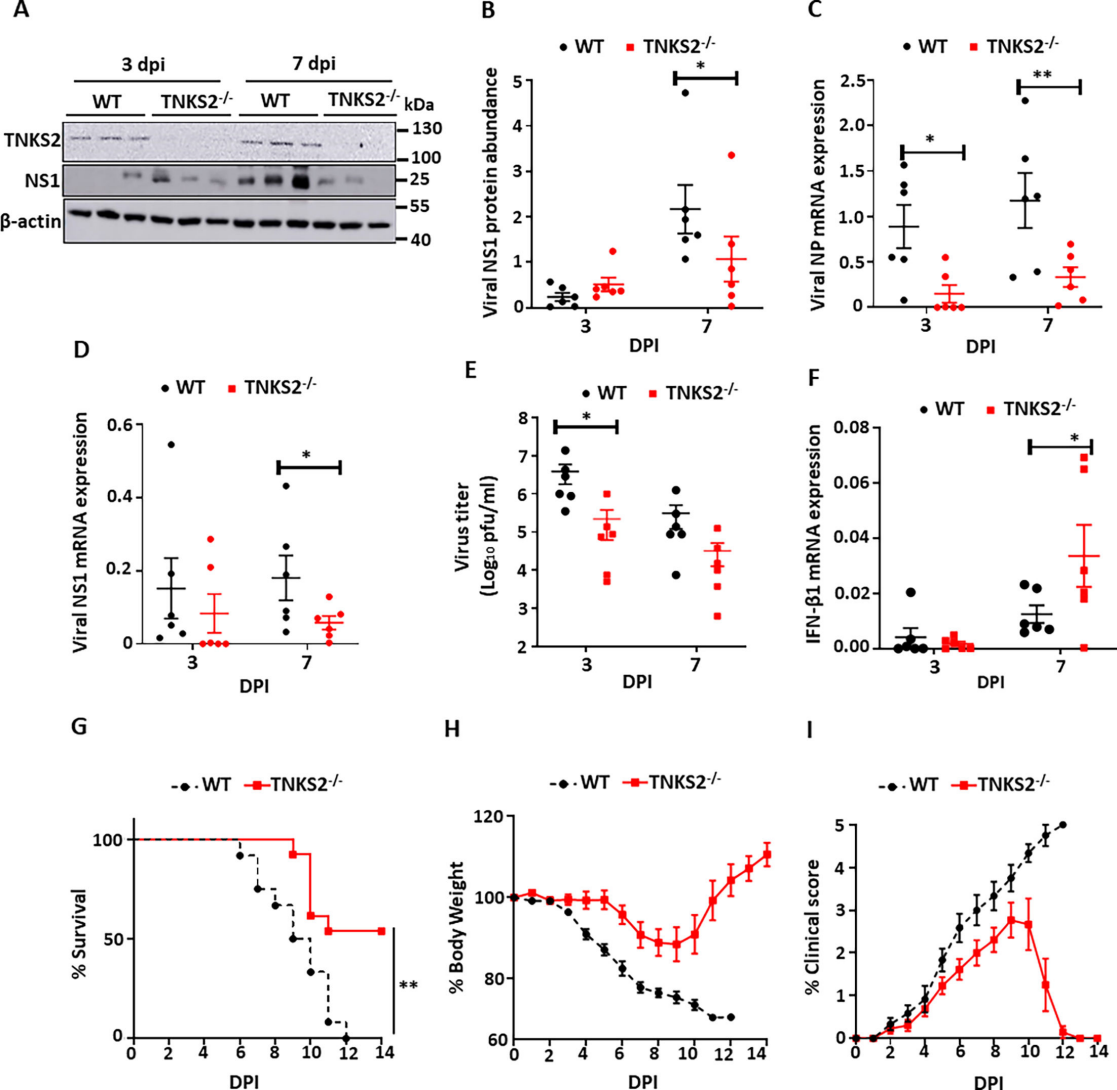
TNKS2 KO in mice attenuates IAV replication and increases survival. (**A–F**) WT and *Tnks2*^-/-^ mice (*N* = 6, 3 males and 3 females per group) were infected intranasally with a sublethal dose of A/PR/8/34 (160 PFU/mouse). Lung tissues were collected at 3 and 7 dpi. TNKS2 and viral NS1 protein expression levels were measured by western blotting and normalized to β-actin (**A, B**). Viral NP and NS1, as well as IFN-β1 mRNA levels, were measured by real-time PCR and normalized to β-actin (**C, D, F**). Virus production in the lungs was measured by plaque assay (**E**). (**G–I**) WT and *Tnks2*^-/-^ mice (*N* = 13, 6 males and 7 females per group) were infected intranasally with a lethal dose of A/PR/8/34 (320 PFU/mouse, 2xMLD_50_). Kaplan-Meier survival analysis of mice (**G**). Body weight loss and clinical scores were determined daily (**H, I**). The results were displayed as the mean ± SE. *P < 0.05, **P < 0.01, vs. WT. (**B–F**) Two-way ANOVA followed by Sidak’s pairwise comparison. (**G**) Mantel-Cox test with Bonferroni-corrected threshold.

Similar studies were performed with *Tnks2*^-/-^ mice. C57BL6/J WT mice were obtained from Jackson laboratories and age- and sex-matched to *Tnks2*^-/-^ mice. The deletion of TNKS1 in *Tnks2*^-/-^ mice was confirmed by western blot (Fig. 8A). Upon a sublethal dose challenge, viral NS1 protein expression was reduced 51 ± 21% at 7 dpi in the lungs of *Tnks2*^-/-^ mice (Fig. 8A and B). Viral NP and NS1 mRNA levels were also decreased at 7 dpi in the lungs of *Tnks2*^-/-^ mice (Fig. 8C and D). Virus production in the lungs was significantly decreased at 3 dpi (Fig. 8E). IFN-β1 mRNA expression was increased at 7 dpi in the lungs of *Tnks2*^-/-^ mice (Fig. 8F). *Tnks2*^-/-^ mice also improved survival against IAV infection and had less body weight loss and a lower clinical score (Fig. 8F through H). Collectively, our data demonstrated that deletion of TNKS1 or TNKS2 in mice suppresses IAV replication and improves survival against a lethal IAV infection.

## DISCUSSION

For many years, two classes of conventional antivirals, M2 ion channel blockers, and neuraminidase inhibitors have been used to suppress influenza virus replication, manage symptoms, minimize hospitalizations, and prevent deaths from severe influenza infections. However, there is growing concern about the emergence of viral resistance to drugs that directly target viral proteins. Thus, there is a need for novel approaches to design and validate effective antiviral drugs that do not develop viral resistance. One of the potential interventions that is being explored is to target host factors that influenza virus relies on to complete its life cycle. Recent development of host-based therapeutics focuses on identifying drugs that prime the host immune system against viral infections and thus achieve a robust and long-lasting effect. In this study, we screened all host PARPs for their effects on influenza virus infection using CRISPR activation and found that TNKS1 and TNKS2 were the most potent PARPs that increase IAV infection. KO of TNKS1 or TNKS2 in cells repressed IAV replication, and TNKS double KO showed the largest effect in limiting IAV replication. TNKS double KO cells showed a more pronounced activation of the type I interferon response compared to WT cells, indicating an enhanced antiviral signaling. Importantly, TNKS1 or TNKS2 KO in mice reduced IAV replication and increased survival against IAV infection.

The influenza virus depends on host cellular machinery for efficient replication and virion production. From influenza virus entry to viral protein assembly and virion budding, cellular host factors play an important role in orchestrating virus life cycle (38). Genome-wide RNAi screening and inhibitor studies for those host factors are of particular importance for current efforts to develop the next generation of anti-influenza drugs (39–41). The finding of those studies demonstrates that cellular host factors can either facilitate or inhibit virus replication. The post-translational modification enzymes, PARPs, can act as pro- or antiviral regulators (7, 42). PARP1, PARP7, PARP11, and PARP14 have been reported to have both pro-viral (14, 43–45) and antiviral (7, 45, 46) roles. Other PARPs, such as PARP9, PARP10, PARP12, and PARP13, are known to antagonize infection (7, 9, 13, 46).

In the present study, CRISPR-mediated activation of PARP1, PARP3, PARP5a, PARP5b, PARP9, and PARP16 led to a significant increase in influenza virus replication. This is consistent with other studies showing that PARP1 plays a pro-viral role in RNA virus infections, including influenza A virus (IAV), Japanese encephalitis virus (JEV), hepatitis C virus (HCV), and porcine reproductive and respiratory syndrome virus (PRRSV) (14, 47–49). However, a recent study on bioRxiv indicated that all PARPs, except for PARP14, exhibited antiviral activity, using an overexpression approach (50). This observation is contrary to our current results using CRISPR activation screen, which shows that many PARPs are pro-viral for influenza virus infection. The reason for the discrepancy is unclear, but it could be due to the different approach for overexpressing PARPs and different assays for influenza virus infection.

Deng and coworkers have shown that TNKS2 interacts directly with Epstein-Barr virus nuclear antigen 1 (EBNA1) to regulate Epstein-Barr virus replication in a PARP-dependent manner (11). Moreover, Li et al. have shown that HSV requires PARP activity of TNKS for its efficient replication. Notably, HSV infection results in the phosphorylation of TNKS1, but not TNKS2 (22). Furthermore, HCMV infection upregulates TNKS1 and TNKS2 expression. Inhibition of TNKS activity or knockdown of TNKS expression promotes the HCMV infection. Additionally, HCMV specifically inhibits TNKS PARP activity, while Axin1 expression is upregulated, and the Wnt/β-catenin pathway is downregulated in the infected cells (10). These previous findings show the positive and negative effects of TNKS on DNA virus replication.

In this study, we have provided both *in vitro* and *in vivo* evidence to support that TNKS1 and TNKS2 are proviral factors that are important for efficient IAV replication. *In vitro*, CRISPR activation of TNKS1 or TNKS2 increased, while CRISPR KO of TNKS1 and/or TNKS2 inhibited IAV infection. Future studies using domain-specific mutants or domain deletion constructs could help elucidate which structural regions of TNKS1/2 are critical for their proviral roles during influenza virus infection. *In vivo*, KO of TNKS1 or TNKS2 reduced IAV replication in the lungs and protected mice against IAV infection. Another research group demonstrated that TNKS2-deficient mice exhibited increased resistance to virus infection (EMCV), while TNKS1-deficient mice showed heightened susceptibility to death induced by EMCV (51).

Mechanistically, our data support that TNKS KO reduces IAV infection by activating type I IFN responses by the following observations: (1) TNKS double KO induced mRNA and protein expression of IFN-β1 during IAV infection, (2) TNKS DKO increased the phosphorylation of Stat1, (3) TNKS DKO enhanced transcriptional activity of Stat1 as demonstrated by an ISRE reporter assay, and (4) TNKS DKO induced the expression of ISGs. Moreover, TNKS1 or TNKS2 KO mice showed the upregulation of IFN-β1 mRNA expression in the IAV-infected lungs. It is noted that whole-body KO mice were used in this study. Mouse lungs have a plethora of cells, such as epithelial cells, fibroblasts, and immune cells. All cells could contribute to type I IFN responses at various levels. Thus, the interpretation of *in vivo* results is more complex than *in vitro* studies.

TNKS is a negative regulator of Axin (36). We have previously shown that overexpression of Axin1 activates JNK/c-Jun signaling and increases type I IFN expression (36). The current study provides evidence that the TNKS KO-mediated increase in type I IFN expression occurs via the Axin1-JNK/c-Jun signaling: (1) Axin1 protein levels were increased in TNKS KO cells and mice, (2) TNKS KO-mediated reduction of IAV infection was abolished by knocking down Axin1, and (3) TNKS DKO activates JNK/c-Jun signaling as demonstrated by the increased phosphorylation of c-Jun and JNK (AP-1) luciferase reporter activity.

In summary, we found that TNKS KO inhibited influenza virus infection *in vitro* and *in vivo* through the activation of antiviral innate immunity induced by type I IFNs. The finding of the current study may contribute to our knowledge on host factor-directed next-generation influenza therapeutics.

## Data Availability

All relevant data supporting the findings of this study are available within the article and its supplemental material. All other data associated with this paper are available upon request from the corresponding author.

## SUPPLEMENTAL MATERIAL

Tables S1 and S2 jvi.01298-25-s0001.docx Primer sequences for real-time PCR and for mouse genotyping.

Table S3 jvi.01298-25-s0002.docx Primer sequences for cloning lenti-sgRNA-MS2-Zeo.

ASM does not own the copyrights to Supplemental Material that may be linked to, or accessed through, an article. The authors have granted ASM a non-exclusive, world-wide license to publish the Supplemental Material files. Please contact the corresponding author directly for reuse.
