# Supplementary material for: Tankyrases positively regulate influenza A virus replication via type I interferon response
Source: J Virol. 2025 Oct 2;99(10):e01298-25. doi: 10.1128/jvi.01298-25 (PMC12548432; doi:10.1128/jvi.01298-25)
Supplement: Tables S1 and S2 — Primer sequences for real-time PCR and for mouse genotyping. [file jvi.01298-25-s0001.docx]

Table 1- Primer sequences for real-time PCR

| **Gene Name** | | **Sequence** |
| --- | --- | --- |
| NP | Forward primer | 5’-TGTGTATGGACCTGCCGTAGC- 3’ |
|  | Reverse primer | 5’-CCATCCACACCAGTTGACTCTTG- 3’ |
| NS1 | Forward primer | 5’-CAAAAGCAGGGTGACAAAGACA- 3’ |
|  | Reverse primer | 5’-CTTGGTCTGCAACTCGTTTGC- 3’ |
| PARP1 | Forward primer | 5’- GGGATGACCAGCAGAAAGTCA -3’ |
|  | Reverse primer | 5’-TACACCCCTTGCACGTACTTC -3’ |
| PARP2 | Forward primer | 5’- GGTGGGGAAGCTCATGTGTAT -3’ |
|  | Reverse primer | 5’-CTGAAGTTCCTCTGGGCATCAT -3’ |
| PARP3 | Forward primer | 5’- TGTTGGCAAGGGCATCTACTT -3’ |
|  | Reverse primer | 5’-CTGGGGTTGTCCGTGTTGATA -3’ |
| PARP4 | Forward primer | 5’- ACTTACCTCAGCAGCAGAAGA -3’ |
|  | Reverse primer | 5’-ACTGACTCAGAACATCAGCAT -3’ |
| TNKS1 | Forward primer | 5’- TGGACGCGGCAAACGTAAAT -3’ |
|  | Reverse primer | 5’-AAGCGGGATGAGACCTCCAT -3’ |
| TNKS2 | Forward primer | 5’- GCTGAGCCAACCATCCGAAAT -3’ |
|  | Reverse primer | 5’-ACTTGCGTGGCAGTTGACA -3’ |
| PARP6 | Forward primer | 5’- ATTTGGGCTGGGTCTTCAGTT -3’ |
|  | Reverse primer | 5’-CCACTTGCCTTGAACCAACTG -3’ |
| PARP7 | Forward primer | 5’- TGATTCTCAGGAGCACTTGGA -3’ |
|  | Reverse primer | 5’-ACAGCCTTCGTAGTTGGTCAA -3’ |
| PARP8 | Forward primer | 5’- ACTATGATGGGGAACTGCACAA -3’ |
|  | Reverse primer | 5’-TGGCTCCATATTCCCTGAGAGA -3’ |
| PARP9 | Forward primer | 5’- GGCAAAGAGGTCCAAGATGCT -3’ |
|  | Reverse primer | 5’-TCCACGTTGAATCCCATCAGA -3’ |
| PARP10 | Forward primer | 5’- CAGAGAACACCGGGGAGTTC -3’ |
|  | Reverse primer | 5’-CTCCCGGTACAGCTCATACTG -3’ |
| PARP11 | Forward primer | 5’- GCCCCCTTTTCTATCAGTGCTT -3’ |
|  | Reverse primer | 5’-TGTGTTTGATTGTGCAGAGGAA -3’ |
| PARP12 | Forward primer | 5’- CCAGGAGGAGGGTGATCAGAT -3’ |
|  | Reverse primer | 5’-CCTCCCATTTGCCTCTATCCA -3’ |
| PARP13 | Forward primer | 5’- CCTGCGATAACCTGCATCTCT -3’ |
|  | Reverse primer | 5’-TGTTCAGTCCAGAGAGTTCGTC -3’ |
| PARP14 | Forward primer | 5’- GTTACCTGCAACCCCAGATGA -3’ |
|  | Reverse primer | 5’-TCCACCATCAAGAGGGAGTTT -3’ |
| PARP15 | Forward primer | 5’- CCAGCTAGAGCCAGGACAATC -3’ |
|  | Reverse primer | 5’-AGCTCTGCCAGAGAAATGCATC -3’ |
| PARP16 | Forward primer | 5’- ATTGACCATCCGGACGTCAAG -3’ |
|  | Reverse primer | 5’-ACTCGCAGCAGCTGGTTATT -3’ |
| Human IFNβ1 | Forward primer | 5’-GCTTGGATTCCTACAAAGAAGCA-3’ |
|  | Reverse primer | 5'ATAGATGGTCAATGCGGCGTC |
| Mouse IFNβ1 | Forward primer | 5’-CAGCTCCAAGAAAGGACGAAC-3’ |
|  | Reverse primer | 5’-GGCAGTGTAACTCTTCTGCAT-3’ |
| Human MX1 | Forward primer | 5’-GTTTCCGAAGTGGACATCGCA-3’ |
|  | Reverse primer | 5’-CTGCACAGGTTGTTCTCAGC-3’ |

Table 2- Primer sequences for mouse genotyping

| **Gene Name** | | **Sequence** |
| --- | --- | --- |
| *TNKS1^-/-^* | WT_Forward primer | 5’-TTT TCA GTT CAG AGT GTG CC-3’ |
|  | WT_Reverse primer | 5’-GTC TCT CTC TGC CCC TTA TC-3’ |
|  | Lox-P (Mut)_forward primer | 5’-TGG CTG GAC GTA AAC TCC TCT TCA GAC CTA ATA AC-3’ |
| *TNKS2^-/-^* | WT_Forward primer | 5'- GCA TAC ACA TCA AAG TTT TCC G -3' |
|  | WT_Reverse primer | 5'- TCT CCT AAC CCC TTT CTC CC -3' |
|  | Mut_forward primer | 5’-GAC GTA AAC TCC TCT TCA GAC GTA ATA AC-3’ |
