# Supplementary material for: Tankyrases positively regulate influenza A virus replication via type I interferon response
Source: J Virol. 2025 Oct 2;99(10):e01298-25. doi: 10.1128/jvi.01298-25 (PMC12548432; doi:10.1128/jvi.01298-25)
Supplement: Table S3 — Primer sequences for cloning lenti-sgRNA-MS2-Zeo. [file jvi.01298-25-s0002.docx]

Table 3- Primer sequences for cloning lenti-sgRNA-MS2-Zeo

| **Gene Name** | | **Promoter**  **Region** | **Chromosome(strand)** | **Start-End** | **Sequence**  **Forward Primer Reverse Primer** | |
| --- | --- | --- | --- | --- | --- | --- |
| PARP1 | sgRNA-1 | Yes | chr1 (-) | 226408275- 226408294 | CACCG**ACAATCAAAGGGGTGGCGCC** | AAACGGCGCCACCCCTTTGATTGTC |
|  | sgRNA-2 | Yes | chr1 (+) | 226408193-226408212 | CACCG**CCTGCCGGGTGGAGCTCTGC** | AAACGCAGAGCTCCACCCGGCAGGC |
|  | sgRNA-3 | Yes | chr1 (+) | 226408150- 22640816 | CACCG**CGCGCGCGCCCCCTGCCGGC** | AAACGCCGGCAGGGGGCGCGCGCGC |
| PARP2 | sgRNA-1 | Yes | chr14 (+) | 20343495-20343514 | CACCG**AGCGACATGCAAATATTGCA** | AAACTGCAATATTTGCATGTCGCTC |
|  | sgRNA-2 | Yes | chr14 (-) | 20343457- 20343476 | CACCG**CCATAAACGTGAAATGTCTT** | AAACAAGACATTTCACGTTTATGGC |
|  | sgRNA-3 | Yes | chr14 (-) | 20343555-20343574 | CACCG**CACCCAGCGCGCGTGCGCCC** | AAACGGGCGCACGCGCGCTGGGTGC |
| PARP3 | sgRNA-1 | Yes | chr3 (-) | 51942196-51942215 | CACCG**AAGGGGCGCAAACACAGAGA** | AAACTCTCTGTGTTTGCGCCCCTTC |
|  | sgRNA-2 | Yes | chr3 (-) | 51942173-51942192 | CACCG**CTCTTGCTCCGACTTCGAAG** | AAACCTTCGAAGTCGGAGCAAGAGC |
|  | sgRNA-3 | Yes | chr3 (-) | 51942249-51942268 | CACCG**GTCACGTTCCAGAACGCGAA** | AAACTTCGCGTTCTGGAACGTGACC |
| PARP4 | sgRNA-1 | Yes | chr13 (-) | 24512954-24512973 | CACCG**ACTGAAAGCCCTGAACCCTC** | AAACGAGGGTTCAGGGCTTTCAGTC |
|  | sgRNA-2 | Yes | chr13 (+) | 24512983-24513002 | CACCG**CAGGAGGGATTTTGTCAATG** | AAACCATTGACAAAATCCCTCCTGC |
|  | sgRNA-3 | Yes | chr13 (-) | 24512901-24512920 | CACCG **CCGGGGACCCGCTCCCCACC** | AAAC GGTGGGGAGCGGGTCCCCGGC |
| PARP5a | sgRNA-1 | Yes | chr8 (-) | 9555837-9555856 | CACCG**AGCGCACAGGAAATGATGCT** | AAACAGCATCATTTCCTGTGCGCTC |
|  | sgRNA-2 | Yes | chr8 (-) | 9555748- 9555767 | CACCG**GGCGGGGCTTTGTCACTGAG** | AAACCTCAGTGACAAAGCCCCGCCC |
|  | sgRNA-3 | Yes | chr8 (+) | 9555881- 9555900 | CACCG**GAAGTGAGGGCGGGCGGTGG** | AAACCCACCGCCCGCCCTCACTTCC |
| PARP5b | sgRNA-1 | Yes | chr10 (+) | 91798198-91798217 | CACCG**ACACAATATGCAGGATCGTT** | AAACAACGATCCTGCATATTGTGTC |
|  | sgRNA-2 | Yes | chr10 (-) | 91798239-91798258 | CACCG**TCGTCCCACCGCCATCTTTG** | AAACCAAAGATGGCGGTGGGACGAC |
|  | sgRNA-3 | Yes | chr10 (-) | 91798264-91798283 | CACCG**GGCTTCGGCGGCGGGAGAAG** | AAACCTTCTCCCGCCGCCGAAGCCC |
| PARP6 | sgRNA-1 | Yes | chr15 (+) | 72271449-72271468 | CACCG**AAAAGGTGAAGAGTTGGAAA** | AAACTTTCCAACTCTTCACCTTTTC |
|  | sgRNA-2 | Yes | chr15 (-) | 72271405-72271424 | CACCG**GGAAAGGGCATTGAGAGTAG** | AAACCTACTCTCAATGCCCTTTCCC |
|  | sgRNA-3 | Yes | chr15 (-) | 72271361-72271380 | CACCG**GCAGCCTGGTTTGAAAAAGC** | AAACGCTTTTTCAAACCAGGCTGCC |
| PARP7 | sgRNA-1 | Yes | chr3 (+) | 156674740- 156674759 | CACCG**CGCGGGCACTGCTTTCCACT** | AAACAGTGGAAAGCAGTGCCCGCGC |
|  | sgRNA-2 | Yes | chr3 (+) | 156674822-156674841 | CACCG**GGGGCTGAAGAACCCTAGCG** | AAACCGCTAGGGTTCTTCAGCCCCC |
|  | sgRNA-3 | Yes | chr3 (-) | 156674776-156674795 | CACCG**TGAACGGGAGCCTGAGAGAA** | AAACTTCTCTCAGGCTCCCGTTCAC |
| PARP8 | sgRNA-1 | Yes | chr8 (-) | 143986562 143986581 | CACCG**AATACCTCCTGGTCAGCTGG** | AAACCCAGCTGACCAGGAGGTATTC |
|  | sgRNA-2 | Yes | chr8 (-) | 143986587 -143986606 | CACCG**GGGTCCTGAGGGGTCCAGCC** | AAACGGCTGGACCCCTCAGGACCCC |
|  | sgRNA-3 | Yes | chr8 (+) | 143986495 -143986514 | CACCG**GGGCCCTGGGCGCTGAGGCC** | AAACGGCCTCAGCGCCCAGGGCCCC |
| PARP9 | sgRNA-1 | Yes | chr3 (+) | 122564374- 122564393 | CACCG**TTTACCGCCCAGCTGCCTCC** | AAACGGAGGCAGCTGGGCGGTAAAC |
|  | sgRNA-2 | Yes | chr3 (+) | 122564778- 122564797 | CACCG**AGTGAACACTCGGTGGAGGA** | AAACTCCTCCACCGAGTGTTCACTC |
|  | sgRNA-3 | Yes | chr3 (+) | 122564801- 122564820 | CACCG**GGCCTCACATGTGCCTGGTG** | AAACCACCAGGCACATGTGAGGCCC |
| PARP10 | sgRNA-1 | Yes | chr8 (-) | 143986562-143986581 | CACCG**AATACCTCCTGGTCAGCTGG** | AAACCCAGCTGACCAGGAGGTATTC |
|  | sgRNA-2 | Yes | chr8 (-) | 143986587-143986606 | CACCG**GGGTCCTGAGGGGTCCAGCC** | AAACGGCTGGACCCCTCAGGACCCC |
|  | sgRNA-3 | Yes | chr8 (+) | 143986495-143986514 | CACCG**GGGCCCTGGGCGCTGAGGCC** | AAACGGCCTCAGCGCCCAGGGCCCC |
| PARP11 | sgRNA-1 | Yes | chr12 (-) | 3873569-3873588 | CACCG**AGATCACTGGGTGATACAGT** | AAACACTGTATCACCCAGTGATCTC |
|  | sgRNA-2 | Yes | chr12 (+) | 3873524-3873543 | CACCG**TGCGAGTCTGTGTCTGACGA** | AAACTCGTCAGACACAGACTCGCAC |
|  | sgRNA-3 | Yes | chr12 (+) | 3873497-3873516 | CACCG**GCGCGGGCGGTCGTCCTGGG** | AAACCCCAGGACGACCGCCCGCGCC |
| PARP12 | sgRNA-1 | Yes | chr7 (+) | 140063824-140063843 | CACCG**GTTACTGGGGGAA CCCTACC** | AAACGGTGGGTTCCCCCAGTAAC |
|  | sgRNA-2 | Yes | chr7 (+) | 140063809-140063828 | CACCG**GTTATGTGTGAAAGTGTTAC** | AAAC GTAACACTTTCACACATAACC |
|  | sgRNA-3 | Yes | chr7 (-) | 140063752-140063771 | CACCG**AGGAAAGATTGCTGGGTCAA** | AAACTTGACCCAGCAATCTTTCCTC |
| PARP13 | sgRNA-1 | Yes | chr7 (-) | 139109851-139109870 | CACCG**AGGCAGAGCGCACGGCTCGC** | AAACGCGAGCCGTGCGCTCTGCCTC |
|  | sgRNA-2 | Yes | chr7 (+) | 139109884-139109903 | CACCG**ATCTTCCCAGCAACTCCACA** | AAACTGTGGAGTTGCTGGGAAGATC |
|  | sgRNA-3 | Yes | chr7 (+) | 139109800-139109819 | CACCG**GTGCGCACCCCACCCATTCC** | AAACGGAATGGGTGGGGTGCGCACC |
| PARP14 | sgRNA-1 | Yes | chr3 (+) | 122680656-122680675 | CACCG**CGCCGCTGGCCAGCCCCAAA** | AAACTTTGGGGCTGGCCAGCGGCGC |
|  | sgRNA-2 | Yes | chr3 (+) | 122680729-122680748 | CACCG**GGGATGACTCTGCCATTCCT** | AAACAGGAATGGCAGAGTCATCCCC |
|  | sgRNA-3 | Yes | chr3 (+) | 122680678-122680697 | CACCG**GTTACTTTGGCCTTGGCTGT** | AAACACAGCCAAGGCCAAAGTAACC |
| PARP15 | sgRNA-1 | Yes | chr3 (+) | 122577483-122577502 | CACCG**AGACCTCAGGATTTTGACCC** | AAACGGGTCAAAATCCTGAGGTCTC |
|  | sgRNA-2 | Yes | chr3 (-) | 122577504-122577523 | CACCG**CGTTACTGCCAGACAAATCC** | AAACGGATTTGTCTGGCAGTAACGC |
|  | sgRNA-3 | Yes | chr3 (+) | 122577439-122577458 | CACCG**TGTACAGATGCTATTGACTG** | AAACCAGTCAATAGCATCTGTACAC |
| PARP16 | sgRNA-1 | Yes | chr15 (+) | 65286809-65286828 | CACCGA**AGCAGCCCCCGGGGGACGG** | AAACCCGTCCCCCGGGGGCTGCTTC |
|  | sgRNA-2 | Yes | chr15 (-) | 65286765-65286784 | CACCGC**ACGTGTCAGCACGTTCCCG** | AAACCGGGAACGTGCTGACACGTGC |
|  | sgRNA-3 | Yes | chr15 (+) | 65286832-65286851 | CACCG**GCAGAGCCCACTCTCCGCGA** | AAACTCGCGGAGAGTGGGCTCTGCC |

The sgRNA sequences used for cloning were previously validated for transcriptional activation using the SAM system, as described in Konermann et al., Nature 2015 (Ref 33), and specifically target the region within 200 bp upstream of the TSS.
